# Severity of nonalcoholic fatty liver disease is associated with subclinical cerebro-cardiovascular atherosclerosis risk in Korean men

**DOI:** 10.1371/journal.pone.0193191

**Published:** 2018-03-22

**Authors:** Jung Eun Lee, Yong Jae Lee, Soo Yoon Chung, Hee Woo Cho, Byoung Jin Park, Dong Hyuk Jung

**Affiliations:** 1 Department of Internal Medicine, Yongin Severance Hospital, Yonsei University College of Medicine, Yongin, Korea; 2 Department of Family Medicine, Gangnam Severance Hospital, Yonsei University College of Medicine, Seoul, Korea; 3 Department of Radiology, Yongin Severance Hospital, Yonsei University College of Medicine, Yongin, Korea; 4 Department of Family Medicine, Yongin Severance Hospital, Yonsei University College of Medicine, Yongin, Korea; University of Kansas Medical Center, UNITED STATES

## Abstract

**Background:**

No studies have reported the relationship between nonalcoholic fatty liver disease (NAFLD) and concurrent cerebral artery and coronary artery atherosclerosis simultaneously. We aimed at determining whether NAFLD, as assessed by ultrasound, is associated with subclinical cerebro-cardio vascular atherosclerosis (CCVA) by multidetector-row computed tomography (MDCT), and high resolution—magnetic resonance angiography (HR-MRA). This cross-sectional study included men in the general Korean population aged 20–70 years.

**Results:**

A total of 1,652 men participated in the study (normal, n = 835; mild-to-moderate NAFLD, n = 512; severe NAFLD, n = 305). The risk of subclinical CCVA was positively associated with age (odds ratio [OR] 1.068; 1.054–1.081, *p* < 0.001), body mass index (OR 1.120; 1.08 0–1.162, *p* < 0.001), hepatic enzyme levels (OR 1.012; 1.001–1.023, *p* = 0.027; OR 1.006; 1.001–1.012, *p* = 0.036), fasting glucose (OR 1.021; 1.015–1.027, *p* < 0.001), triglycerides (OR 1.002; 1.000–1.003, *p* = 0.016), hypertension (OR 2.836; 2.268–3.546, *p* < 0.001), and diabetes (OR 2.911; 2.137–3.964, *p* < 0.001). Also, high-density lipoprotein cholesterol was inversely associated with subclinical CCVA (OR 0.974; 0.965–0.982, *p* < 0.001). Compared with normal controls, the OR for subclinical CCVA after full adjustment was 1.46 in the mild-to-moderate NAFLD group (95% confidence interval [CI]: 1.10 to 1.93) and 2.04 in the severe NAFLD group (95% CI: 1.44 to 2.89).

**Conclusions:**

Our data show that NAFLD is common among Korean men, and NAFLD severity on ultrasonography is associated with subclinical CCVA, as assessed by MDCT, and HR-MRA.

## Introduction

Nonalcoholic fatty liver disease (NAFLD) comprises a spectrum of liver conditions ranging from simple hepatic steatosis to liver cirrhosis. Hepatic steatosis is the most common liver disease in developed countries, with a prevalence of 15%-30% in the general population and 70%-90% in obese individuals or those with type 2 diabetes [1–3]. Although fatty liver has long been considered a benign condition, several studies have shown that hepatic steatosis is a feature of metabolic syndrome [4,5] and is related to abdominal obesity, insulin resistance, type 2 diabetes, hypertension, inflammation, and dyslipidemia [6–8]. Recently, hepatic steatosis has been investigated as a potential risk factor for atherosclerosis. A previous study reported that hepatic steatosis is not only a marker of vascular atherosclerosis but may be related to its pathogenesis [9]. Several recent studies reported that patients with fatty liver have an increased risk of cardiovascular disease, including coronary artery disease and stroke [10,11].

Previous studies demonstrating an association between NAFLD and cardiovascular disease focused on coronary artery calcification or plaque, typically assessed by carotid intima-media thickness (CIMT) [12,13]. No published studies have evaluated the relationship between NAFLD and concurrent coronary artery and cerebral artery atherosclerosis.

Therefore, in the present study we evaluated the relationship between NAFLD and coronary artery atherosclerosis, as assessed by multidetector-row computed tomography (MDCT), a noninvasive method that can detect coronary plaque with high accuracy and examine the anatomy of coronary arteries to predict the severity of coronary artery disease. We also investigated the relationship between NAFLD and cerebral artery atherosclerosis, as assessed by high-resolution magnetic resonance imaging (HR-MRI) and magnetic resonance angiography (MRA). In addition, we evaluated the relationship between NAFLD severity, as assessed by ultrasonography, and subclinical cerebro-cardiovascular atherosclerosis (CCVA) in Korean men.

## Materials and methods

### Study population

**Fig 1:**
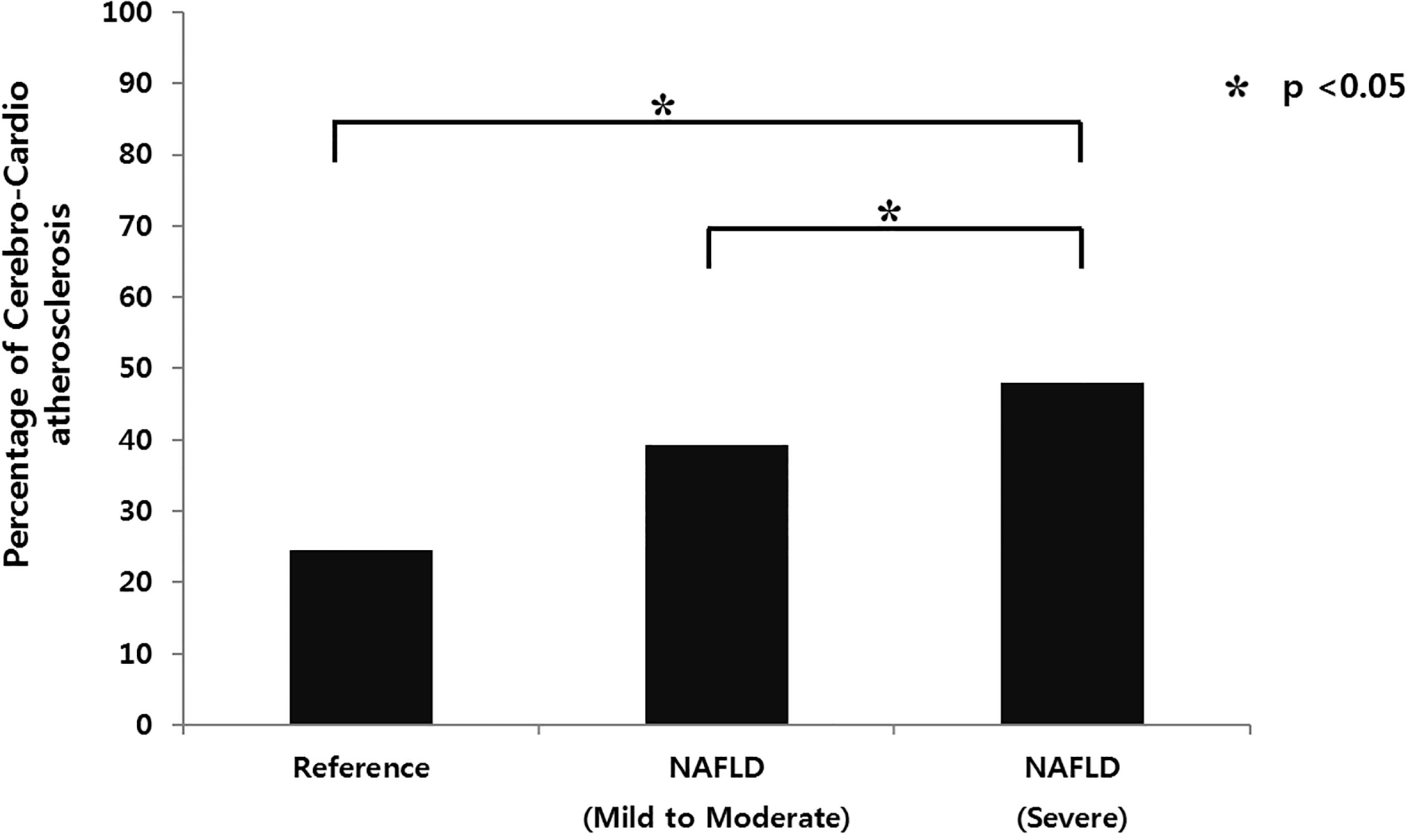
Prevalence of subclinical cerebro-cardio vascular atherosclerosis increased with the severity of NAFLD.

A total of 1799 men, aged 20 or older, who underwent a routine health examination, were enrolled at the health promotion center of Gangnam Severance Hospital in Seoul, Korea between January 2007 and July 2010. Individuals meeting any of the following criteria were excluded: missing covariate information (n = 19), alcohol intake ≥ 140 g/week (n = 68), a positive test for hepatitis B antigens or hepatitis C antibodies (n = 18), or history of cancer (n = 10) or cardiovascular disease (n = 32). The remaining 1652 individuals were included in the final analysis (Fig 1). The study protocol conformed to the ethical guidelines of the Declaration of Helsinki and was approved by the institutional review board at Gangnam Severance Hospital, Yonsei University College of Medicine, Seoul, Korea (no. 3-2016-0051). The need for informed consent was waived owing to the retrospective nature of the study and the data were analyzed anonymously.

### Definitions

We defined subclinical CCVA as positive findings on brain and neck HR-MRI, MRA, or MDCT. Cerebral atherosclerosis was defined as brain MRI and MRA findings of any stenosis in cerebral, carotid and vertebral arteries. We excluded total occlusion of these arteries in order to rule out the possibility of embolization due to atrial fibrillation or cardiac valvular vegetation.

Degree of coronary atherosclerosis was assessed by MDCT and categorized as insignificant or significant stenosis. Significant luminal narrowing was defined as lumen diameter reduced by≥50% compared to normal areas of the stenotic coronary arteries (proximal or distal to the stenotic lesions).

### Data collection

All participants completed a health questionnaire and underwent a routine physical examination. Body weight and height were measured while the participants were wearing light indoor clothing without shoes. After the participants had rested for at least 5 minutes in a quiet room, blood pressure (BP) was measured using a standard mercury sphygmomanometer (Baumanometer, Copiague, NY). With the participant seated, an appropriately-sized cuff (based on mid-arm circumference) was placed snugly around the upper right arm at the heart level. Two measurements were made at ≥ 5-minute intervals, and the mean of the two measurements was used in the analyses. Hypertension was defined as systolic BP ≥140 mmHg, diastolic BP ≥90 mmHg, and/or previous use of antihypertensive medication. Smoking status was based on self-report. Never smokers were defined as participants who had smoked <100 cigarettes (<5 packs of cigarettes) in their lifetime. Current smokers were defined as those who had smoked ≥100 cigarettes in their lifetime and reported “currently smoking” in the questionnaire. Former smokers were defined as those who had smoked ≥100 cigarettes in their lifetime but reported “abstain from smoking” in the questionnaire.

A venous blood sample was drawn after the participants had fasted for ≥12 hours or overnight. Fasting plasma glucose level was determined by a glucose oxidase-based assay. Diabetes mellitus was defined as fasting plasma glucose ≥126 mg/dL and/or use of a hypoglycemic agent or insulin. Serum concentrations of aspartate aminotransferase (AST), alanine aminotransferase (ALT), total cholesterol, high-density lipoprotein cholesterol (HDL-C), low-density lipoprotein cholesterol (LDL-C) and triglycerides (TG) were determined using the Hitachi 7600–110 automated chemistry analyzer (Hitachi, Tokyo, Japan). We obtained the hepatic steatosis index (HSI) for the accuracy of the degree of NAFLD measured by ultrasound. HSI is a simple, efficient screening tool for NAFLD that may be utilized for selecting individuals for liver ultrasonography [14]. We calculated the HSI as follows:

HSI=8×ALT/AST+BMI+Sex(female=2,male=0)+DM(yes=2,no=0)

### Ultrasound examination

The diagnosis of fatty liver was based on abdominal ultrasonography with a 3.5-MHz transducer (HDI 5000, Philips, Bothell, WA, USA) performed by two experienced radiologists who were unaware of the aim of the study. The diagnosis of fatty liver was made based on the presence of diffusely increased liver echogenicity with evident contrast between the liver and the kidney, diffusely increased liver echogenicity with blurring of the intrahepatic vessels or the diaphragm, or bright liver echogenicity with poor penetration of the posterior hepatic segment and intrahepatic vessels or invisibility of the diaphragm [15]. In other words, the normal liver parenchyma has a homogeneous echotexture with echogenicity equal to or slightly greater than that of the renal cortex and spleen. When the echogenicity is just increased, it is mild fatty liver; when the echogenic liver obscures the echogenic walls of portal vein branches, it is moderate, and, when the echogenic liver obscures the diaphragmatic outline, it is grade severe fatty liver [16]. Liver with any degree of fat accumulation was considered NAFLD in the present study. We excluded individuals with evidence of secondary causes of NAFLD, such as viruses or autoimmune disorders.

### MDCT examination

Computed tomography coronary angiography was performed using a 64-MDCT scanner (Philips Brilliance 64, Philips Medical System, Best, Netherlands). For patients who had a heart rate ≥ 65 beats per minute, a β-blocker (40–80 mg propranolol; Pranol, Dae Woong, Seoul, Korea) was administered orally 1 hour before examination to decrease the heart rate. We used prospective electrocardiographic gating with a step-and-shoot technique and additional padding of the tube-on time. With the patient in the supine position, MDCT was performed in the craniocaudal direction within a single breath-hold at the end-inspiratory suspension. Iodinated contrast medium (1.0 mL/kg, Optiray 350; Tyco Healthcare, Kanata, Canada) was administered intravenously at a rate of 5 mL/s using a power injector (Nemoto Kyorindo, Tokyo, Japan).

Imaging was performed using real-time bolus tracking with the region of interest in the ascending aorta. The scans were started 7 s after reaching a trigger threshold of 110 Hounsfield units. Participants held their breath during imaging. The scanning parameters were as follows: step-and-shoot axial scanning, 420-ms gantry rotation time, 120 kV, 210 mAs, 64×0.625 mm slice collimation, and 4 cm table feed per rotation. Image reconstruction was performed on the scanner’s workstation using commercially available software (Extended Brilliance Workstation, Philips Medical System, Best, Netherlands). Original axial images and multiplanar reformatted reconstructions rendered orthogonal and perpendicular to the vessel course of each respective segment were used for assessment. Curved multiplanar reformation images were generated.

### HR MRI and MRA examination

All MRI examinations were performed on a 3.0 T MRI system (Signa 3.0T, General Electric, Milwaukee, WI, USA; Achieva 3.0T, Philips Medical Systems, Best, Netherlands). Diffusion-weighted images were obtained using the following parameters: TR/TE 9000/81.4 ms, matrix 128× 128, FOV 240×240 mm, slice thickness 4 mm, b = 1000 s/mm^2^. The voxel volumes were 14 mm^3^. Three-dimensional brain MRA was performed for arterial and venous phase imaging with following parameters: repetition time 4900 ms, echo time minimum, a 30×27 cm field of view, image matrix 256×192, flip angle 20°, and section thickness 14 mm. The dose of gadobutrol (Gadovist^®^; Schering, Berlin, Germany) was 0.2 mL/kg of body weight. The contrast agent injected with a power injector (Spectris MR Injector^®^; Medrad, Pennsylvania, USA) at a rate of 2 mL/sec. When the contrast was visually detected in the carotid artery by the technologist on axial plane during injection of the contrast, the coronal 3D gradient echo sequence was initiated; and image acquisition time 2–3 minutes. After acquisition of arterial phase of MRA, venous phase images were obtained. Major brain and neck artery atherosclerosis was assessed on MRA using picture archiving communication system technology (PACS; GE Medical Systems, Milwaukee, WI, USA).

### Statistical analysis

Participant characteristics were summarized according to group (normal, mild-to-moderate NAFLD, or severe NAFLD) using one-way analysis of variance (ANOVA) for continuous variables and chi-square test for categorical variables. Odds ratios (ORs) and 95% confidence intervals (CIs) for the risk of subclinical CCVA were calculated using multiple logistic regression analysis after adjusting for confounding factors. All data were analyzed using SPSS for Windows Version 20.0 (IBM Corp., Armonk, NY, USA).

## Results

The demographic and clinical characteristics of the 1652 study participants are shown according to NAFLD severity in Table 1. Of the 817(49.5%) patients with NAFLD, 512 (62.7%) had mild-to- moderate NAFLD, and 305 (37.3%) had severe NAFLD, as diagnosed by abdominal ultrasound. The mean age of the participants was 53.1 years, and mean body mass index (BMI) was 24.1 kg/m^2^. The groups differed significantly (*p* < 0.001) across all categories except age (p = 0.145). Compared to normal controls, subclinical CCVA was more prevalent among participants with NAFLD, as was diabetes, hypertension, and smoking. Other important clinical differences between groups included BMI and serum levels of AST, ALT, gamma glutamyl transferase (ɤGT), total cholesterol, TG, HDL-C, LDL-C and fasting glucose.

**Table 1:** Baseline characteristics of the study population according to nonalcoholic fatty liver severity, as assessed by ultrasound.

	normal (n = 835)	mild to moderate NAFLD(n = 835)	severe NAFLD(n = 305)	p-value
Age (years)	53.2±9.0	54.1±9.4	53.0±8.8	0.145
BMI (kg/m^2^)	22.9±2.6	25.0±2.4	26.2±2.9	< 0.001
AST (IU/L)	21.9±7.6	24.2±9.3	28.2±12.1	< 0.001
ALT (IU/L)	21.4±10.4	29.3±21.6	37.8±21.8	< 0.001
ɤGT (IU/L)	35.6±57.1	47.9±67.4	48.8±37.1	< 0.001
Fasting glucose (mg/dL)	95.4±20.5	101.4±18.4	105.2±24.8	< 0.001
Total-cholesterol (mg/dL)	188.7±33.9	191.7±35.2	202.7±36.5	< 0.001
Triglyceride (mg/dL)	105.1±56.9	140.5±74.4	180.6±109.5	< 0.001
HDL-C (mg/dL)	52.8±13.6	45.5±10.3	43.3±8.7	< 0.001
LDL-C(mg/dL)	117.8±30.5	121.5±32.0	130.1±34.0	< 0.001
Smoking status, n (%)				
Never	343(41.1)	136(26.5)	80(26.2)	< 0.001
Current	212(25.4)	160(31.3)	92(30.2)	
Ex	280(33.5)	216(42.2)	133(43.6)	
Systolic BP (mmHg)	122.7±16.5	127.6±16.4	130.4±16.1	< 0.001
Diastolic BP (mmHg)	76.3±9.5	80.0±9.9	81.5±8.8	< 0.001
Hypertension, n (%)^a^	203(24.4)	158(30.8)	126(41.3)	< 0.001
Diabetes, n (%)^b^	65(7.8)	73(14.2)	56(18.4)	< 0.001
Hepatic steatosis index ^c^	30.8±4.0	34.75±4.5	37.2±4.4	< 0.001
Subclinical CCVA, n (%)	205(24.5)	201(39.3)	146(47.9)	< 0.001

All data are presented as mean ± SD, proportions, or medians (interquartile ranges) for skewed variables.

*p* value was calculated by one-way ANOVA for continuous variables and chi-square test for categorical variables.

^a^ Hypertension was defined as a systolic BP ≥ 140 mmHg, diastolic BP ≥ 90 mmHg, or previous use of antihypertensive medication.

^b^ Diabetes was defined as fasting plasma glucose level ≥126 mg/dL or use of hypoglycemic agent or insulin.

^C^ Hepatic steatosis index was calculated as: 8 * ALT/AST + BMI(+2, if diabetes; +2, if female)

NAFLD, nonalcoholic fatty liver disease; BMI, body mass index; AST, aspartate aminotransferase; ALT, alanine aminotransferase; ɤGT, gamma glutamyl transferase; BP, blood pressure; CCVA, cerebro-cardiovascular atherosclerosis.

Table 2 shows the result of univariate analysis to assess the relationship between subclinical CCVA and clinical variables. The risk of subclinical CCVA was positively associated with age (OR 1.068; 1.054–1.081, *p* < 0.001), BMI (OR 1.120; 1.080–1.162, *p* < 0.001), systolic BP (OR 1.022; 1.015–1.028, *p* < 0.001), diastolic BP (OR 1.034; 1.023–1.046, *p* < 0.001), hypertension (OR 2.836; 2.268–3.546, *p* < 0.001), diabetes (OR 2.911; 2.137–3.964, *p* < 0.001), and levels of AST (OR 1.012; 1.001–1.023, *p* = 0.027), ALT (OR 1.006; 1.001–1.012, *p* = 0.036), fasting glucose (OR 1.021; 1.015–1.027, *p* < 0.001), and triglycerides (OR 1.002; 1.000–1.003, *p* = 0.016). HDL-C was inversely associated with subclinical CCVA (OR 0.974; 0.965–0.982, *p* < 0.001). In addition, the prevalence of subclinical CCVA increased with the severity of NAFLD (Fig 1).

**Table 2:** Results of univariate analyses to assess the relationship between clinical variables and subclinical cerebro-cardiovascular atherosclerosis (CCVA).

Variable	Subclinical CCVA	
	Odds ratio	*p* value
Age (years)	1.068 (1.054–1.081)	< 0.001
BMI (kg/m^2^)	1.120 (1.080–1.162)	< 0.001
AST (IU/L)	1.012 (1.001–1.023)	0.027
ALT (IU/L)	1.006 (1.001–1.012)	0.036
ɤGT (IU/L)	1.002 (1.000–1.004)	0.061
Fasting glucose (mg/dl)	1.021 (1.015–1.027)	< 0.001
Total cholesterol (mg/dl)	0.997 (0.965–1.001)	0.074
Triglyceride (mg/dl)	1.002 (1.000–1.003)	0.016
HDL-C (mg/dl)	0.974 (0.965–0.982)	< 0.001
Systolic BP (mmHg)	1.022 (1.015–1.028)	< 0.001
Diastolic BP (mmHg)	1.034 (1.023–1.046)	< 0.001
Hypertension (%)	2.836 (2.268–3.546)	< 0.001
Diabetes (%)	2.911 (2.137–3.964)	< 0.001

BMI, body mass index; AST, aspartate aminotransferase; ALT, alanine aminotransferase; ɤGT, gamma-glutamyl transferase; HDL-C, high density lipoprotein cholesterol; BP, blood pressure.

Table 3 shows the results of logistic regression analysis to determine whether NAFLD severity was independently associated with subclinical CCVA. Our results showed that NAFLD severity was associated with a higher risk for subclinical CCVA. After adjusting for age and BMI, the OR for subclinical CCVA in the mild-to-moderate NAFLD group was 1.67 (95% CI: 1.30 to 2.21) compared with normal controls. After full adjustment (adjusting for age, BMI, diabetes, hypertension, smoking, AST, ALT, ɤGT, fasting glucose, total cholesterol, triglycerides, HDL-C, systolic BP, and diastolic BP), the OR for subclinical CCVA in the mild-to-moderate group was 1.46 (95% CI: 1.10 to 1.93) compared with normal controls. The OR for subclinical CCVA was significantly higher for participants with severe NAFLD after adjusting for age and BMI (2.43, 95% CI: 1.76 to 3.34) and after full adjustment (2.04, 95% CI: 1.44 to 2.89).

**Table 3:** Odds ratios and 95% confidence intervals for risk of subclinical cerebro-cardio vascular atherosclerosis according to severity of nonalcoholic fatty liver disease (NAFLD) in adult men.

	Reference	mild to moderate NAFLD	*p*-value	severe NAFLD	*p*-value
Model 1^a^	1.00	1.67 (1.30–2.21)	0.001	2.43 (1.76–3.34)	0.001
Model 2^b^	1.00	1.55 (1.18–2.04)	0.002	2.13 (1.53–2.96)	0.001
Model 3^c^	1.00	1.46 (1.10–1.93)	0.006	2.04 (1.44–2.89)	0.001

^a^ Model 1: adjusted for age and body mass index (BMI).

^b^ Model 2: adjusted for age, BMI, diabetes, hypertension, and smoking.

^c^ Model 3: adjusted for age, BMI, diabetes, hypertension, smoking, systolic blood pressure, diastolic blood pressure, and levels of aspartate aminotransferase, alanine aminotransferase, gamma-glutamyl transferase, fasting glucose, total cholesterol, triglycerides, and HDL-cholesterol.

## Discussion

In this study we found that NAFLD was associated with subclinical CCVA in the asymptomatic general population of Korean men. Higher prevalence of subclinical CCVA was observed in patients with NAFLD as compared to controls without NAFLD. In addition, we found that severe NAFLD was associated with a higher risk of large-vessel atherosclerosis. This finding is consistent with data showing that severity of histological features of NAFLD is independently associated with increasing carotid artery atherosclerosis, as assessed by CIMT [17]. Several previous studies have described a relationship between NAFLD and cardiovascular disease [18,19]; however, the small sample size of these studies makes it difficult to draw conclusions. To the best of our knowledge, this is the first study that shows an association between NAFLD and concurrent subclinical CCVA through the use of highly accurate imaging studies, performed simultaneously. Several epidemiological studies have also reported that NAFLD is associated with an increased risk of cardiovascular disease, independent of traditional cardiometabolic risk factors [5,18]. Liu et al. suggested that NAFLD was a strong predictor of cardiovascular disease and may play a pivotal role in the development of atherosclerosis [19].

Although the precise mechanism linking vascular atherosclerosis and NAFLD is ulclear, the following factors have been proposed as possible mechanisms: insulin resistance, inflammation, oxidative stress, and endothelial dysfunction [20–25]. The pathogenesis of NAFLD is linked to insulin resistance, with hepatic fat accumulation as one of the best predictors of insulin resistance [19]. High insulin levels leads to increase liver glucose production, thereby exacerbating steatogenesis. Furthermore, excess caloric intake and increasing hepatic lipogenesis inhibit fatty acid oxidation and decrease the removal of lipids from hepatic circulation [21]. Therefore, hepatic steatosis may be an important factor in whole-body insulin resistance and dyslipidemia throughout overproduction of cholesterol-rich remnant particles, leading to atherosclerosis. Our results cannot determine whether there is a direct association between insulin resistance and NAFLD because we did not evaluate insulin resistance using homeostatic model assessment. However, NAFLD was associated with BMI, TG, and HDL cholesterol by univariate analysis, all of which are related to insulin resistance, suggesting that a direct association may exist.

The association of NAFLD with inflammation and oxidative stress may also partially explain the increased risk of atherosclerosis. Inflammation plays a pivotal role in the pathogenesis of NAFLD [22,23]. Leach et al. reported that patients diagnosed with hepatic steatosis by liver biopsy have higher homocysteine levels than healthy controls [24], and a cross-sectional study conducted by Ajmal et al. showed that NAFLD is associated with increased C-reactive protein (CRP) [25]. Both oxidative stress and inflammation are important in the progression from simple steatosis to steatohepatitis [26]. Although it is unclear whether hepatic steatosis causes atherosclerosis directly through inflammation and reactive oxygen species, indirectly though insulin resistance-related processes, or both, the risk of atherosclerosis is higher with severe fatty liver than with simple steatosis. In this study, the results of multiple logistic regression analysis also showed a graded association between subclinical CCVA and the severity of hepatic steatosis. However, it is not possible to draw the conclusion that NAFLD is directly associated with inflammation, because we did not measure inflammatory markers such as CRP or tumor necrosis factor-α (TNF-α) due to the limits of the study design. This disadvantage should be addressed by a prospective study in the future.

Although a systematic review and meta-analysis confirmed the strong association between NAFLD and increased CIMT [27], McKimmie et al. suggested that hepatic steatosis may not be a direct mediator of cardiovascular disease, and Petit et al. concluded that fatty liver is not independently associated with CIMT in patients with type 2 diabetes [28,29]. Most previous studies used CIMT to assess carotid artery atherosclerosis [9,17]; however, the variability of CIMT values limits its ability to predict the extent and severity of coronary artery disease [30]. In this study we used brain MRI and MRA imaging for a more accurate assessment of carotid artery stenosis. Also, to accurately determine the severity of coronary artery disease, we used MDCT, another noninvasive imaging method. Although NAFLD has been shown to be associated with coronary artery calcification, as assessed by MDCT [31,32], few studies have reported a direct association between NAFLD and coronary artery stenosis. We evaluated coronary artery stenosis by MDCT instead of the calcium score for a more accurate assessment of coronary artery stenosis. Our study confirmed the findings of Idilman et al., who reported that NAFLD was associated with significant coronary artery disease in 273 patients with type 2 diabetes assessed by MDCT [11]. Consequently, our report firmly confirms the relationship between NAFLD and coronary artery disease, in which our study include more number of patients compared than their findings [11].

The current study has several limitations. First, this study design was cross-sectional study. So, the causal relationship between hepatic steatosis and subclinical CCVA could not be precisely identified. Also, we did not evaluate cardiovascular events or mortality. Second, because liver biopsy is invasive, we assessed NAFLD by ultrasound, which is a highly operator-dependent technique. Although ultrasonography may lead to an incorrect diagnosis of NAFLD in 10%-30% of cases [33], this technique has advantages such as safety, and repeatability, and specificity [34]. Third, we did not routinely check antinuclear antibodies to rule out autoimmune hepatitis; however, the prevalence of autoimmune hepatitis is very low in Korea. Also, we did measure hepatitis B virus antigen and hepatitis C virus antibodies so that we could exclude patients with hepatitis B or C viral infections, which are endemic in South Korea. Finally, we did not evaluate inflammatory markers, such as CRP and TNF-α, because these subjects were healthcare group. Despite of these limitations, our study strengths include the large sample size and utilization of MDCT, brain HR-MRI, and MRA, which provide highly accurate diagnostic information. Consequently, the graded association between NAFLD severity, as assessed by ultrasound, and atherosclerosis, as assessed by MDCT or MRA, confirms the strong association between carotid atherosclerosis and severity of NAFLD.

In conclusion, our results indicate that NAFLD is common in the general population of Korean men and is closely associated with subclinical CCVA, as assessed by MDCT or MRA, regardless of the presence of hypertension or diabetes. In particular, the severity of NAFLD on ultrasound was an independent predictor for subclinical CCVA, which is an independent risk factor for cardiovascular outcome. Accordingly, we recommend that physicians actively monitor for the development of cardiovascular disease.
